# Immersive Virtual Reality and Vestibular Rehabilitation in Multiple Sclerosis: Case Report

**DOI:** 10.2196/31020

**Published:** 2022-02-16

**Authors:** Cristina García-Muñoz, María-Dolores Cortés-Vega, Juan-Carlos Hernández-Rodríguez, Lourdes M Fernández-Seguín, Isabel Escobio-Prieto, María Jesús Casuso-Holgado

**Affiliations:** 1 Department of Physiotherapy Faculty of Nursing, Physiotherapy and Podiatry University of Seville Seville Spain; 2 Department of Dermatology Hospital Universitario Virgen del Rocío Seville Spain

**Keywords:** immersive virtual reality, vestibular rehabilitation, multiple sclerosis, exergames

## Abstract

**Background:**

Dizziness and imbalance are common and disabling symptoms in patients with multiple sclerosis (MS) and are caused by a central, peripheral, or mixed vestibulopathy. Central vestibular disorder is the most frequently reported vestibular problem in the MS population due to demyelination. Vestibular rehabilitation ameliorates these symptoms and their repercussions and improves quality of life. Immersive virtual reality (VRi) is an emerging tool in this field; however, no previous research has been performed studying its effects in MS.

**Objective:**

The aim of this study was to apply a VRi vestibular training protocol to a patient with MS and assess the effects induced by the experimental intervention.

**Methods:**

This case study included a 54-year-old woman with relapsing-remitting MS. We developed a standardized VRi exercise protocol for vestibular rehabilitation based on the gold-standard Cawthorne-Cooksey vestibular training protocol. The 20-session intervention was made up of 10 initial sessions and 10 advanced sessions. Each 50-minute session was performed two to three times per week for 7 weeks. Four evaluations were carried out over the study period: at baseline (T0), between initial and advances phases (T1), postintervention (T2), and 1 month after the experimental procedure (T3). The research outcomes were dizziness, balance, gait, impact of fatigue, quality of life, repercussions in muscular tone, and usability of the head-mounted display device.

**Results:**

After implementing the VRi vestibular protocol, improvements were seen in the following patient parameters: Dizziness Handicap Inventory score (62 points at T0; 4 points at T2); Berg Balance Scale score (47 points at T0; 54 points at T2); instrumented Timed Up and Go time (8.35 seconds at T0; 5.57 seconds at T2); muscular tone of the erector spinae, rectus femoris, and soleus; Modified Fatigue Impact Scale score (61 points at T0; 37 points at T2); and Multiple Sclerosis Quality of Life-54 values (67.16% in the physical health area at T2; 33.56% in the mental health area at T2). The patient rated the usability of the system as 90%, based on the System Usability Scale, and gave the system a grade of A.

**Conclusions:**

Although further research is needed, this study provided initial evidence that the first VRi vestibular protocol for the MS population can improve dizziness, balance, gait, impact of fatigue, quality of life, and muscular tone through an exergame intervention. This study may help establish a standardized VRi protocol for vestibular rehabilitation.

## Introduction

Relapsing-remitting multiple sclerosis (RRMS) is the most frequent phenotype of multiple sclerosis (MS) and is characterized by relapse or attacks [1,2]. Among the symptoms or sequelae of relapse are vertigo, dizziness, and postural balance disorders [3]. Vertigo is defined as a rotative illusion that can affect 20% to 50% of patients with MS along the disease course [4,5]. Dizziness is usually accompanied by balance problems and affects 49% to 59% of patients with MS [6]. Central, peripheral, or mixed vestibular disorders are possible etiologies in patients with MS, which explain the presence of dizziness, vertigo, and postural disorders [5,7,8]. In spite of the peripheral affection of the vestibular system being quite common in MS, central vestibular disorder is the most common due to the demyelination process in MS [8,9]. The scientific literature has reported that the MS population could benefit from the effects on dizziness and postural balance of vestibular rehabilitation, whether the vestibular lesion is central, peripheral, or a combination of the two [10]. Vestibular rehabilitation involves exercises targeted to improve vertigo, dizziness, and imbalance and its repercussions during basic activities of daily living [11]. Vestibular exercises are performed to train the vestibulo-ocular reflex (VOR) and vestibulo-spinal reflex (VSR), promoting the neuroplastic mechanisms of adaptation, habituation, and substitution [12,13]. Adaptation will train the VOR through head and ocular movements. Habituation aims to eliminate dizziness symptoms by exposing the subject to several environments and repetitive exercises. Substitution compensates for vestibular deficits through visual or somatosensory systems [14,15].

An emerging tool within vestibular rehabilitation and neurorehabilitation is immersive virtual reality (VRi) [16,17], which immerses patients through a head-mounted display (HMD) in a 360° virtual environment and enables interactions for achieving a specific objective [18]. Some of the advantages of VRi in rehabilitation are the direct auditory and visual feedback provided to the patient, multitasking, a wide variety of environments, and the sense of presence and immersion [18-21]. Exergames are a combination of exercises and video games; these are the principal choice of intervention in VRi for improving the physical condition of users [22,23]. Exergames provide task-oriented training, motivation, and distraction during exercise [22,24]. A recent systematic review reported these additional clinical benefits from VRi compared to conventional vestibular rehabilitation [25]. The same study supported the need for designing a standardized VRi vestibular intervention protocol [25-27].

Regarding the absence of a VRi vestibular exercise program for the MS population and the need to achieve a standardized VRi protocol for vestibular rehabilitation, we chose to design and develop both a program and a protocol. To the best of our knowledge, this is the first VRi vestibular exercise protocol for the MS population. Thus, the primary goal of research was to apply this innovative intervention and evaluate its effects on dizziness and balance in a patient with RRMS. Changes in gait parameters, the impact of fatigue, quality of life, muscular tone repercussion, and usability were assessed as secondary objectives.

## Methods

### Ethics Statements

Ethical approval was provided by the Regional Ethical Review Board in Andalucía, Spain, on March 25, 2020 (ID 2148-N-19). Before recruitment, the participant was provided with written and oral information. Informed consent, which adhered to the principles of the Declaration of Helsinki, was given by the subject in order to be included in the experimental intervention.

### Outcomes and Measurements

Four evaluations of the following five outcomes were performed to detect changes in the patient: dizziness, postural balance, gait parameters, fatigue impact, and quality of life. These assessments were carried out at baseline (T0), between initial and advanced phases (T1), postintervention (T2), and 1 month after the experimental procedure (T3). After VRi, the vestibular protocol usability of the Oculus Quest HMD (Facebook Technologies) was measured using the System Usability Scale (SUS) and a semistructured interview.

### Patient Information

The participant was a 54-year-old woman who was diagnosed with RRMS in 2013 by an expert neurologist and met the McDonald diagnostic criterion. The patient’s Mini-Mental State Examination score was 25 out of 30 due to a memory impairment caused by an MS attack. Her Expanded Disability Status Scale score was 3.0 out of 10.0, which indicated that ambulation was preserved without a walking aid. In 2019, she suffered from an MS attack combined with vertigo and nausea, which lasted more than 24 hours and was unassociated with a specific postural position of onset. Furthermore, in a posterior magnetic resonance imaging (MRI) scan, demyelinating lesions were observed on the right lateral margin of the fourth ventricle, where vestibular nuclei are located [28]. This MRI finding could be related to the vertigo episode. Considering the duration and characteristics of the vertigo episode, along with a negative semicircular canal affection dismissed by the Dix-Hallpike maneuver and Miniconi test, a central vestibular disorder was confirmed. At the initial evaluation, the participant reported severe dizziness (62/100 points) as assessed by the Dizziness Handicap Inventory (DHI) [29,30], accompanied by postural problems and a reluctance to move her head while walking or performing abrupt cephalic movements. Balance examination was carried out through the Berg Balance Scale (BBS) [31], where the participant obtained a total of 47 out of 56 points. It is necessary to highlight the existence of a pronounced imbalance in three specific conditions of the scale: Romberg with closed eyes, tandem position, and single-leg support. The patient was unable to reach or maintain the first two conditions, and she was only able to stand for less than 10 seconds during the last one. A combined analysis by inertial sensors—myoMOTION inertial sensors and software (Noraxon)—and the instrumented Timed Up and Go (iTUG) test [32,33] was carried out to determine the baseline gait parameters of the patient with RRMS. The iTUG global time was 8.35 seconds; the iTUG times for the first and second 180° turns were 0.90 seconds and 0.69 seconds, respectively. Additionally, the sit-to-stand transition time was 1.20 seconds, and the stand-to-sit transition time was 1.03 seconds. The mean gait speed was 1.2 km per hour, and the step cadence was 106 steps per minute. The complete analysis of the gait parameter data is shown in the Results section. The patient’s perceived fatigue impact was 61 out of 84 points on the Modified Fatigue Impact Scale (MFIS) [34]. Quality of life before the experimental intervention was measured using the Multiple Sclerosis Quality of Life-54 (MSQoL-54) [35]; the patient obtained a result of 45.62% for physical health and 25.75% for mental health. Bilateral muscular tone assessment in the erector spinae, the rectus femoris, and the soleus was performed using the MyotonPRO digital palpation device (Myoton AS) [36] during standing after vestibular stimulation through the Miniconi test [37]. All baseline data are listed in detail in the Results section.

### Materials and Intervention

**Figure 1:**
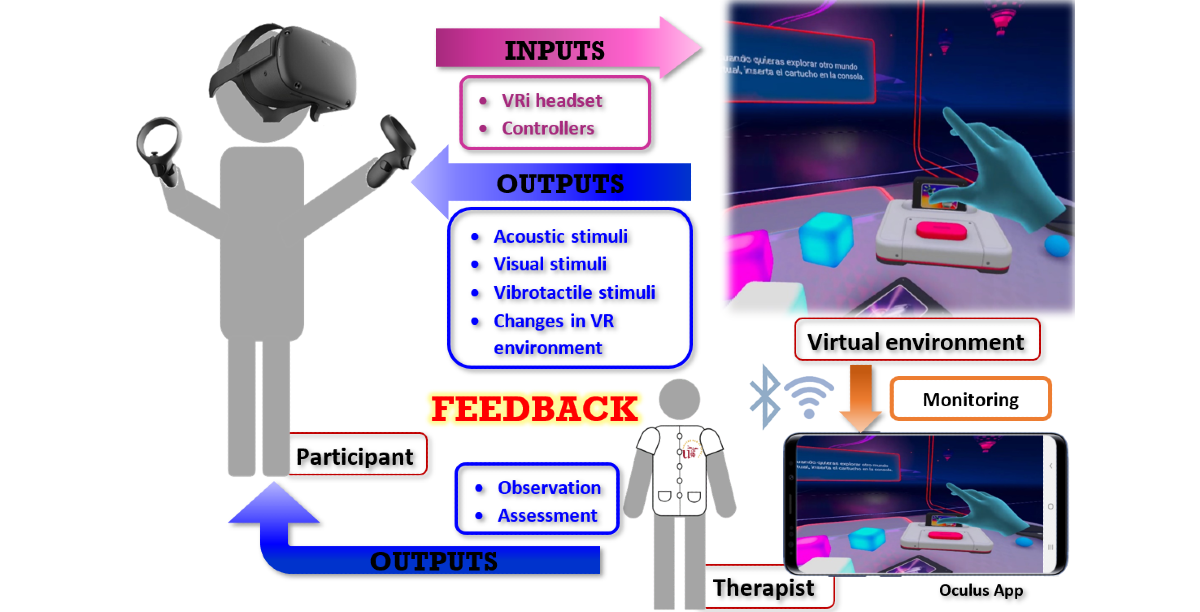
Inputs and outputs in immersive virtual reality (VRi) systems.

The Oculus Quest is a wireless VRi device with high-quality graphics that is economically affordable, as compared to other options on the market [38,39]. VRi systems work via an input and output flow (Figure 1). Inputs are recorded by a VRi headset and controllers in response to external actions of the participant; these inputs then induce changes in the virtual environment through the software. Outputs are changes in the virtual environment providing the subject with a multisensory stimulation source (ie, visual, acoustic, and vibrotactile information). Minimal requisites to start interacting with the Oculus virtual environment include a space greater than 2 × 2 meters to ensure a safe play area and a Wi-Fi connection. The experimental intervention was carried out at the participant’s home and was supervised by a therapist.

**Figure 2:**
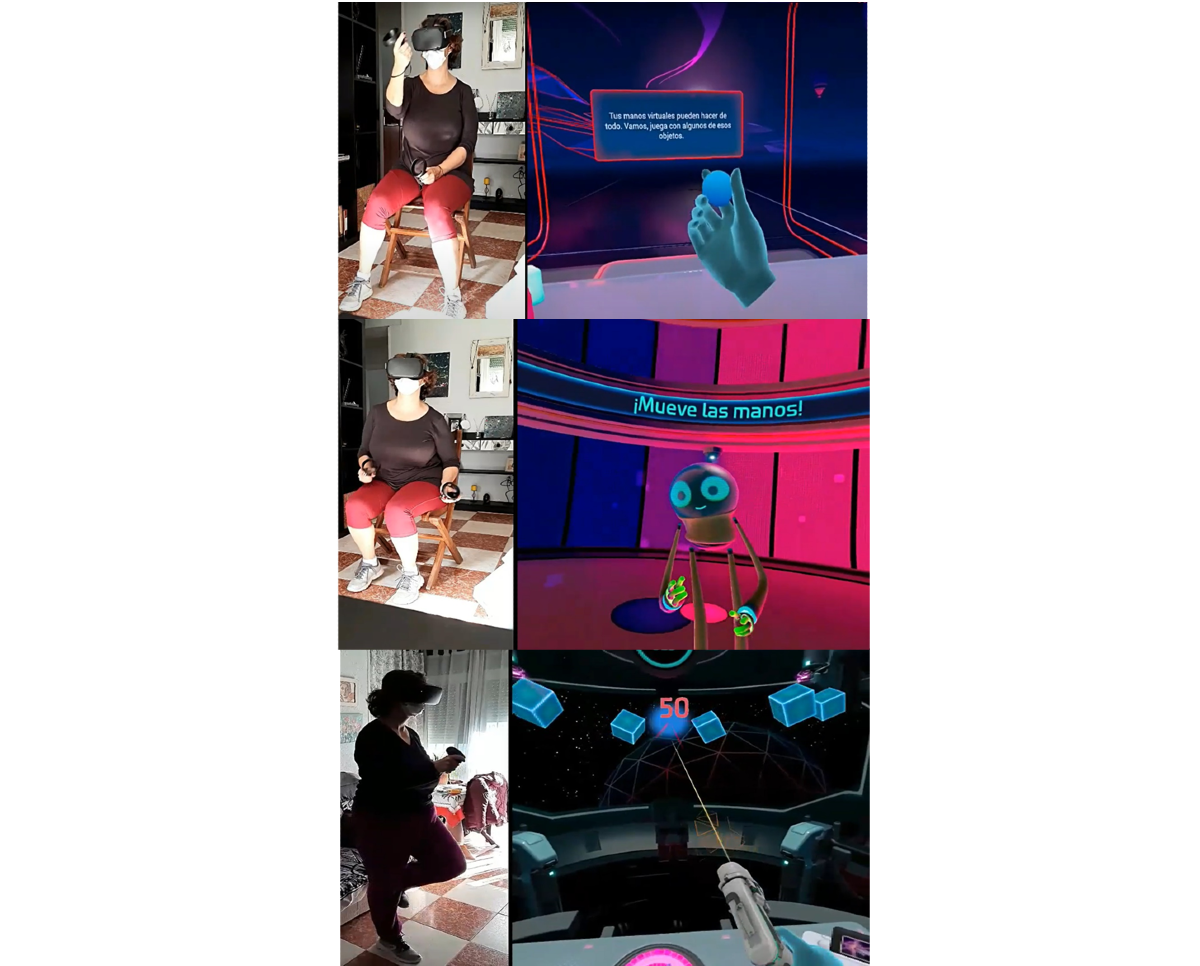
Study participant interacting with First Steps virtual environments. Main room of First Steps (top); Dance with Robot (middle); Shots in Space (bottom).

**Figure 3:**
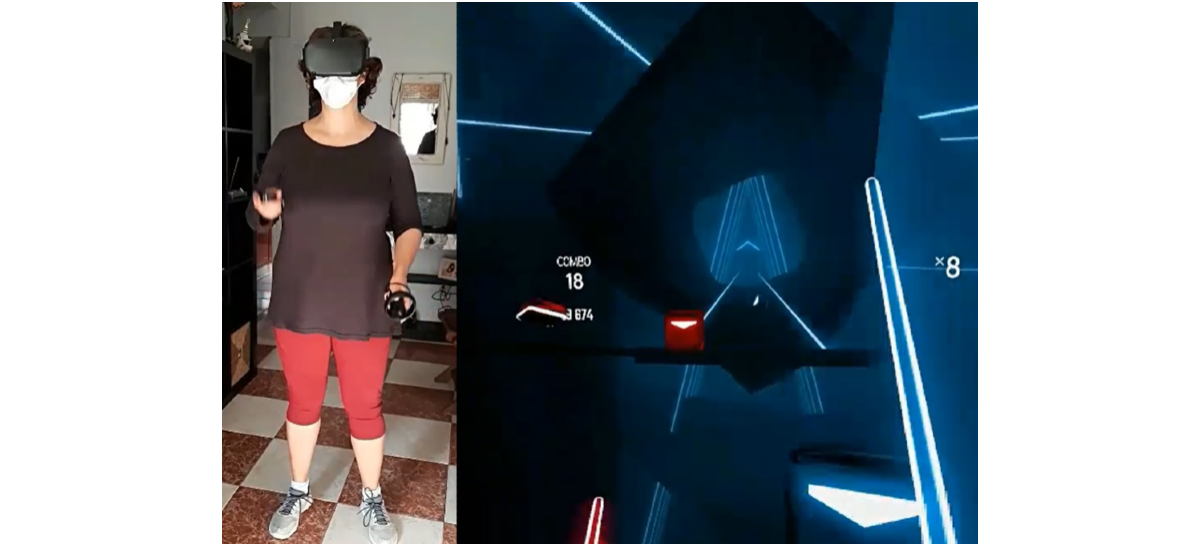
Study participant interacting with Beat Saber environment.

**Figure 4:**
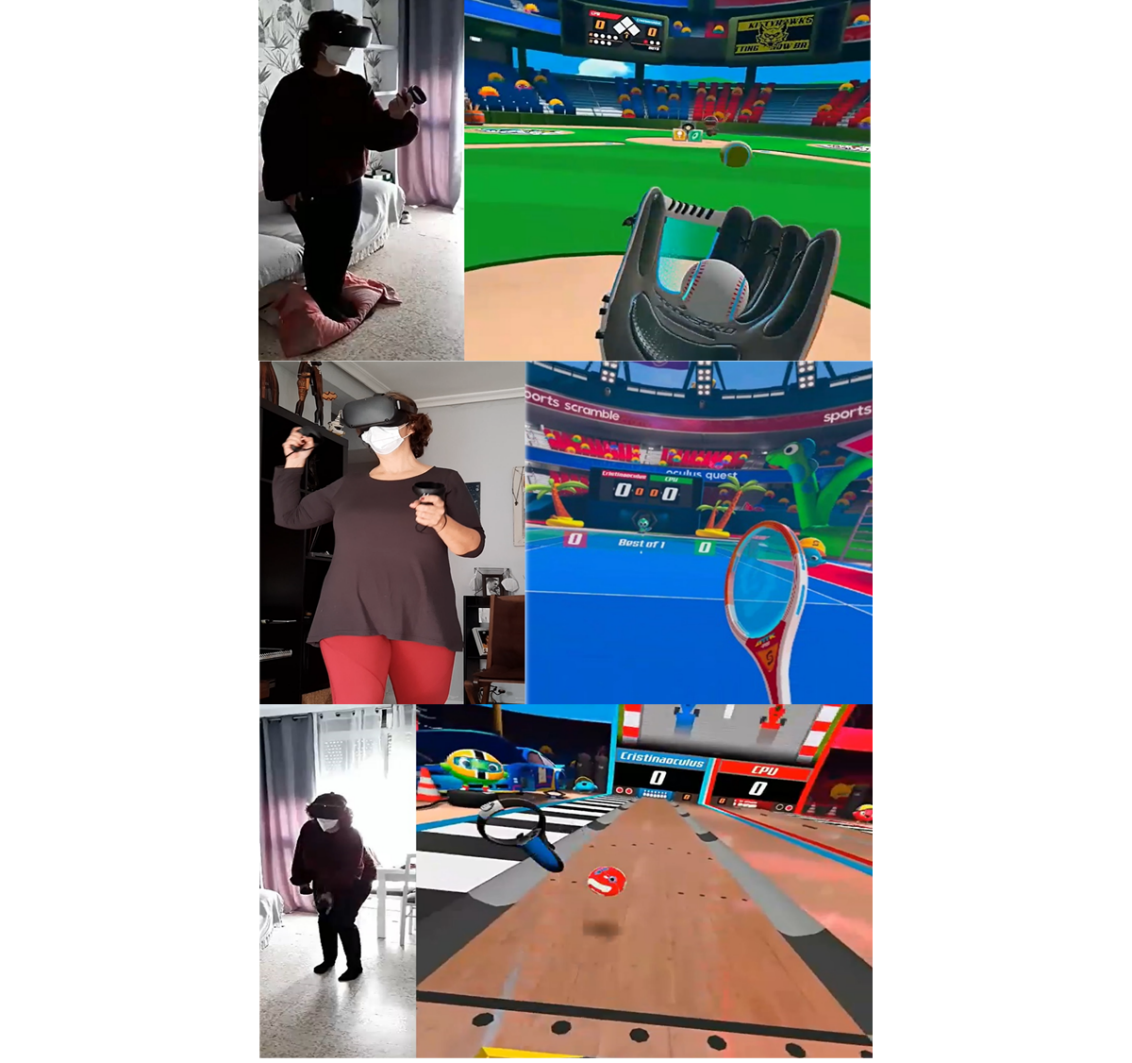
Study participant interacting with Sports Scramble virtual environments. Baseball (top); tennis (middle); bowling (bottom).

Selected VRi exergames are freely available in the Oculus App and include the following: First Steps, Beat Saber, and Sports Scramble. The environments in First Steps include the main room of First Steps, Dance with Robot, and Shots in Space. The first one is a room where the subject can interact with virtual objects (cubes, paper planes, etc). In Dance with Robot, the patient must dance while following some orders. Finally, Shots in Space is a shooter game in which the participant shoots random targets that move over a spatial station (Figure 2). Beat Saber is a rhythm game in which blocks are cut in a specific direction with sabers (Figure 3). Sports Scramble is a sports game where the user can play tennis, baseball, and bowling, with funny virtual elements (eg, a cheese ball instead of a bowling ball; Figure 4). The complete VRi vestibular intervention was carried out over 20 sessions, which occurred two to three times per week over 7 weeks. Each session lasted for 50 minutes. The VRi vestibular protocol can be divided into initial and advanced phases of 10 sessions each. All above-mentioned vestibular exercises were designed to enhance the neuroplastic mechanisms of adaptation, habituation, and substitution and to train the user’s VOR and VSR. Also, to design and create vestibular exercises for this new VRi vestibular protocol, we considered the gold-standard vestibular training from Cooksey [40] as well as key points from Han et al [12] and Whitney and Sparto [13]. Each session from both phases consisted of 15 exercises, which were performed from the easiest to the most complex. Therefore, this gradual exposure of the patient to exercises during sessions prevents the development of dizziness and cybersickness. Exercises and the duration of both phases are described in Table 1. The initial phase consisted of three blocks of exercises, two of which were done seated and the last of which was done in the standing position. Exercises in the advanced phase were performed by disturbing the somatosensory system, reducing the support base, using alternating single-leg support, adopting the tandem position, or adding an unstable surface. This was done to enhance the participation of the vestibular system in maintaining postural balance by the substitution mechanism. New vestibular parameters were added in this second phase of the intervention with quicker head and eye movements, sit-to-stand transitions or vice versa, and 360° turns.

**Table 1:** Exercises performed as part of the immersive virtual reality vestibular intervention.

Phases and exercise instructions					Virtual environments			Duration			Repetitions		
**Initial phase: 50 minutes (45 min of intervention + 5 min of rest)**													
	**Before rest time: 24 minutes (all First Steps environments and Beat Saber)**												
		Take the ping-pong ball and put it in front of your face and move it closer and farther	Main room of First Steps			11 minutes^a^			10 slow repetitions, then 10 fast repetitions				
		Move an object in front of your eyes and follow it; shoot targets that appear in the exergame	Main room and Shots in Space (First Steps)			Main room: 11 minutes; Shots in Space: 7 minutes^b^			Main room: 10 slow repetitions, then 10 fast repetitions; Shots in Space: 1 repetition for each gun				
		Shoot targets that appear randomly inside the virtual environment	Shots in Space (First Steps)			7 minutes			1 repetition for each gun				
		Cut blocks with a saber while your head is fixed; hit a ball in the main room and fixate your gaze on its movement while your head is fixed	Beat Saber and main room of First Steps			Beat Saber: 3 minutes; main room: 11 minutes			Beat Saber: 1 repetition; main room: 10 slow repetitions, then 10 fast repetitions				
		Take a block from the virtual desk, bring it to the floor, and then move it above your head, while staring at it	Main room of First Steps			11 minutes			10 slow repetitions, then 10 fast repetitions				
		Shrink your shoulders while dancing with a robot	Dance with Robot (First Steps)			3 minutes			1 repetition				
		Bend forward and move a virtual block between your knees	Main room of First Steps			11 minutes			10 slow repetitions, then 10 fast repetitions				
	**After rest time: 21 minutes (Beat Saber and Sports Scramble)**												
		Sit down and stand up, and vice versa, with your eyes open	Beat Saber			3 minutes			1 repetition				
		Stand up and move to the right while standing	Bowling (Sports Scramble)			6 minutes^c^			3 repetitions				
		Stand up and move to the right or the left while taking a bowling ball	Bowling (Sports Scramble)			6 minutes			3 repetitions				
		Throw or hit a ball in front of your face	Baseball and tennis (Sports Scramble)			Baseball: 8 minutes; tennis: 4 minutes			Baseball: 1 repetition; tennis: 1 repetition				
		Throw the ball to hit the bowling pins under knee level	Bowling (Sports Scramble)			6 minutes			3 repetitions				
**Advanced phase: 50 minutes (45 min of intervention + 5 min of rest)**													
	**Before rest time: 24 minutes (main room of First Steps)**												
		Take a block from the virtual desk and when you stand up, throw it at a virtual sign situated inside the virtual environment		Main room of First Steps			2 minutes			10 repetitions			
		Move a virtual block at eye level; take a virtual block and throw it from one hand to the other		Main room of First Steps			5 minutes			10 repetitions moving the object, then 10 repetitions throwing the object			
		Take a virtual block, turn 360°, and throw the block to a located target in the environment		Main room of First Steps			5 minutes			10 repetitions to the right, then 10 repetitions to the left			
		In a standing position with a narrow base of support, hit a ball and follow its movements with your head		Main room of First Steps			2 minutes			5 repetitions (eg, 1 repetition lasts until the ball stops)			
		Take the ping-pong ball and put it in front of your face, then move it closer and farther away		Main room of First Steps			5 minutes			10 slow repetitions, then 10 fast repetitions			
		Take the ping-pong racket and hit blocks to one side and the other, following them with your head		Main room of First Steps			2 minutes			15 repetitions			
		Take a virtual block from the desk, then move it to the floor and bring it above your head while standing on a foam surface		Main room of First Steps			3 minutes			10 repetitions			
	**After rest time: 21 minutes (Shots in Space in First Steps, Beat Saber, and Sports Scramble)**												
		Shoot targets with a single gun while supported on a single leg, then the other		Shots in Space (First Steps)			2 minutes			1 repetition			
		Shoot targets with a double gun while maintaining a tandem position		Shots in Space (First Steps)			2 minutes			1 repetition			
		Shoot targets with a machine gun while standing on a foam surface		Shots in Space (First Steps)			2 minutes			1 repetition			
		Hit and cut blocks in a specific direction with sabers while standing on a foam surface; the head is fixed while you make ocular movements		Beat Saber			3 minutes			1 repetition			
		Throw the ball in a baseball stadium while standing on a foam surface		Baseball (Sports Scramble)			4 minutes			1 repetition			
		Bowl with your feet together		Bowling (Sports Scramble)			2 minutes			1 repetition			
		Bowl while standing on a foam surface		Bowling (Sports Scramble)			2 minutes			1 repetition			
		Walk down a bowling alley while moving your head from side to side and the throw the bowling ball		Bowling (Sports Scramble)			4 minutes			2 repetitions			

^a^Within this set of exercises, all exercises in the main room of the First Steps environment combined were performed in 11 minutes.

^b^Within this set of exercises, all Shots in Space exercises combined were performed in 7 minutes.

^c^Within this set of exercises, all bowling exercises in Sports Scramble combined were performed in 6 minutes.

## Results

All outcome results at T1, T2, and T3, as compared to T0, are shown in Table 2.

Regarding baseline dizziness that evolved from a severe condition (DHI score = 62 points) as compared to the absence of it after the combined VRi vestibular protocol, there was a reduction of 58 points on the DHI (T2 DHI score = 4 points). This improvement in dizziness examined by the DHI continued for 1 month after the end of the VRi sessions.

Between phases of the experimental intervention, no changes were assessed for postural balance, but balance impairments improved postintervention. The participant’s BBS score reached a maximum of 56 points at T3. Pathological conditions of Romberg with closed eyes, unstable single-leg support, and the inability to stay in the tandem position were reverted by achieving stable balance.

The participant ameliorated their global iTUG time by 2.35 seconds after the intervention, adding 0.43 seconds to this time 1 month after the VRi program. The rest of the iTUG parameters (ie, sit-to-stand transition and vice versa and both 180° turns) showed a remarkable reduction, as observed in Table 2. Stance and swing phase equated to 50% in both feet, while double support time was 291 milliseconds less in T2 compared to baseline. The minor stride time and the higher stride length were recorded in T3 as 862 milliseconds and 63 centimeters, respectively. The speed of gait and step cadences increased by 1.4 km per hour and 45 steps per minute in T3 from T0, respectively.

Regarding fatigue impact of the subject, a score of 38 was considered when determining the difference between fatigued and nonfatigued [41]. The MFIS score reached 35 points in T1 and 37 points in T2, which reflected a nonfatigued perception of the patient, but this improvement was not maintained 1 month after the intervention ended (MFIS score = 47 points).

The study participant experienced a gain in physical health of 21.54%, as measured by the MSQoL-54, when baseline and postintervention data were assessed. Then, 1 month later, her physical quality of life reached 69.44%, and her mental health reached 42.79%.

**Table 2:** Results and changes in study outcomes at the four measurement points.

Outcome (measurement)		Measurement point			
		Baseline (T0)	Between initial and advanced phases (T1)	Postintervention (T2)	1 month after the experimental procedure (T3)
**Dizziness (Dizziness Handicap Inventory^a^), points**					
	Global	62	26	4	6
	Physical	20	18	4	6
	Emotional	14	2	0	0
	Functional	28	6	0	0
**Postural control (Berg Balance Scale^b^), points**					
	Global	47	47	54	56
**Spaciotemporal parameters of gait (instrumented Timed Up and Go test)**					
	Total time, seconds	8.35	7.00	6.00	5.57
	Sedestation to bipedestation time, seconds	1.20	0.97	0.95	0.77
	First turn, seconds	0.90	0.91	0.79	0.78
	Second turn, seconds	0.69	0.61	0.58	0.50
	Bipedestation to sedestation time, seconds	1.03	0.64	0.58	0.50
	Stance phase, left foot, %	70.8	71.7	68.8	51.1
	Stance phase, right foot, %	70.9	66.3	65.5	49.6
	Swing phase, left foot, %	29.2	28.3	31.2	48.9
	Swing phase, right foot, %	29.1	33.7	34.5	50.4
	Double support time, ms	631	329	340	79
	Stride length, cm	40	46	50	63
	Velocity, km/h	1.2	1.6	1.9	2.6
	Stride time, ms	1205	1031	941	862
	Step cadence, steps/min	106	129	298	151
**Impact of fatigue (Modified Fatigue Impact Scale^c^), points**					
	Global	61	35	37	47
	Physical effort	21	8	7	15
	Cognoscitive effort	36	24	29	30
	Psychosocial effort	4	3	1	2
**Quality of life (Multiple Sclerosis Quality of Life-54), %**					
	Physical health area	45.62	53.14	67.16	69.44
	Mental health area	25.75	36.15	33.56	42.79
**Muscular tone (MyotonPRO^d^), Hz**					
	Right erector spinae	13.0	12.7	13.1	12.3
	Left erector spinae	12.9	12.3	11.7	11.6
	Right rectus femoris	13.9	12.4	13.5	12.4
	Left rectus femoris	13.7	11.8	14.4	12.5
	Right soleus	26.3	23.7	21.9	21.7
	Left soleus	24.0	21.4	20.6	19.4
Usability (System Usability Scale), %		N/A^e^	N/A	90^f^	N/A

^a^Dizziness Handicap Inventory scores range from 0 to 100 for the global scale; the physical subscale ranges from 0 to 28; the emotional and functional subscales range from 0 to 36.

^b^Berg Balance Scale scores range from 0 to 56: 41-56 (low fall risk); 21-40 (medium fall risk); 0-20 (high fall risk).

^c^Modified Fatigue Impact Scale scores range from 0 to 84; the physical effort subscale ranges from 0 to 36; the cognoscitive effort subscale ranges from 0 to 40; the psychosocial effort subscale ranges from 0 to 8.

^d^The MyotonPRO takes three consecutive measurements and reports the final value as the mean; however, the SD is not reported.

^e^N/A: not applicable; the System Usability Scale was only administered at T2.

^f^A 90% value on the System Usability Scale represents a grade of A.

A reduced trend for muscular tone was found in the MyotonPRO data for the three examined muscles. In some cases, there were disparities, primarily in the left erector spinae and bilateral rectus femoris at T2. However, all mean results for the erector spinae, the rectus femoris, and the soleus were lower at T3 compared to T0. The tone of the erector spinae was 19.2% lower for the right side and 21.2% lower for the left side when compared to baseline. Also, 1 month after the experimental intervention, decreases in tone compared to the baseline data were recorded in the rectus femoris (T3 right: –11.4%; left: –9.2%) and in the soleus (T3 right: –19.2%; left: –21.2%).

Postintervention (T2) usability of the Oculus Quest device and perceived satisfaction with the experimental intervention was assessed using the SUS and a semistructured interview. A SUS score of 90% for usability and a grade of A were marked by the participant with RRMS for the Oculus Quest headset. During the interview process, the following impressions were reported by the patient:

I enjoyed the intervention through the virtual device so much. I would continue with it.

These kinds of physiotherapy sessions are more motivating than usual ones in which I get bored really soon because exercises are repetitive.

I have improved my postural balance a lot; now I can take a shower with my eyes closed without falling or I can put my pants on without sitting down.

## Discussion

### Principal Findings

The VRi vestibular training protocol was successfully performed by a participant with RRMS, improving their basal conditions of dizziness, postural balance, gait, fatigue, quality of life, and muscular tone repercussion after the intervention.

HMDs have been described as proper tools with which to apply vestibular rehabilitation by previous studies [16,39,40]. One of the reasons why VRi devices have become a suitable option is because of their accurate tracking systems that record movements by gyroscopes, magnetometers, and accelerometers in six degrees of freedom [42]. Likewise, the control of movement, visual information, and changes in the virtual environment are broken down into cephalic movements, just as the information is provided by the vestibular system [42,43]. Furthermore, thanks to the characteristics of these devices, the neuroplastic mechanisms by which the vestibular system recovers can be trained. Among them, habituation stands out due to the large number of environments to which the subject is exposed and because of the possibility of performing repetitive exercises in a motivational way [12,13].

Improvements in dizziness conditions measured by the DHI have been reported before by Micarreli et al [44] and Viziano et al [45] in peripheral vestibular impairments after combined VRi and vestibular rehabilitation. In these studies, the HMD intervention was implemented using a smartphone; however, commercial HMDs, such as the Oculus Quest, present higher usability and graphic quality compared to these devices [46,47]. Both groups of researchers implemented home-based virtual reality (VR) vestibular programs combined with conventional vestibular therapy in their experimental groups to ensure adherence to treatment. The adherence and security related to these home-based exercise VR programs should be studied deeply [48]. Even though the selected VR devices were wireless and portable, the home-based intervention was discarded. Despite the intervention being performed at the subject’s home due to their physiological characteristics (ie, imbalance and dizziness) and progression of exercises (unstable surface, tandem, etc), the therapist needed to be next to the patient to prevent falls. Also, due to her memory problems, adherence to treatment was not guaranteed, which also required monitoring, as described by Micarreli et al [44] and Viziano et al [45].

Moreover, dizziness is related to imbalance or postural problems, like positive Romberg with closed eyes, unstable single-leg support, or difficulty in the tandem position. Equally, dizziness during cephalic movements while walking is one of the main clinical manifestations in vestibular disorders [49]. Global postural control ameliorated after the VRi intervention was conducted. BBS scores increased by 7 points when comparing measurements at T2 with those at T0, and the maximum score was reached 1 month later by the participant. Once the intervention finished, the patient with RRMS was able to stand in the eyes-closed Romberg position and maintain single-leg support and the tandem position for more than 30 seconds. Ozkul et al [50] confirmed better results in postural balance after an experimental intervention with the Oculus Quest HMD after 16 sessions, as compared to conventional balance training. However, this author did not examine vestibular rehabilitation effects; reported results were similar to those obtained in this case report. In the vestibular framework, Yeh et al [51] and Hsu et al [52] reported better balance performance assessed by posturography in Meniérè disease (ie, peripheral disorder) after a VR vestibular intervention that combined Wii, Kinect, a smartphone HMD, and big screens. Better balance and dizziness were reported by Hsu et al [52] in VR groups compared to groups performing Cawthorne-Cooksey traditional exercises (*P*<.001).

To the best of our knowledge, this is the first study to examine gait parameters in a subject with MS to evaluate changes caused by vestibular training. Gait parameter assessment was carried out because of its remarkable role during walking [53]. The higher gait speed and step cadence during the iTUG test seen in this case study could be related to enhancement of the postural balance of the subject, due to a better vestibular function [54]. A reduction in double stance time could also be explained by the greater performance of the vestibular system [55]. Other studies based on vestibular training for peripheral vestibular problems support the gait data we collected during the iTUG test for global time [56,57]. According to Witchel et al [58], a lower sit-to-stand transition time could be related to greater balance performance, as reported in our case study. Additionally, a vestibular intervention has been shown to be effective in the achievement of a shorter time to perform 180° turns as measured by the iTUG test [59]. In this case study, there was a reduction of 0.11 seconds in both turns after the experimental intervention.

Fatigue is one of the most disabling symptoms among patients with MS and can contribute to postural disorders or a worse performance in the iTUG test [60,61]. In this case, better results in the iTUG test and balance could be related to lower fatigue impact. Vestibular rehabilitation and VR interventions have shown positive effects on fatigue impact in people with MS [50,62]. Dizziness, postural balance impairments, and fatigue are considered the most disturbing symptoms affecting patients’ quality of life [63]. Therefore, after improvement in the above-mentioned symptoms, an increase in quality of life, as measured by the MSQoL-54, was registered in this participant; this was also seen in Ozgen et al [56]. In the Ozgen et al study, after 16 sessions (20 minutes per session) of vestibular balance and ambulation exercises to ameliorate vestibular disturbances in a sample of patients with MS, an improvement in quality of life was recorded within the group, as compared to no intervention (*P*<.001) [56].

Regarding muscular tone repercussion after VRi vestibular training, disparities obtained in the left erector spinae and rectus femoris at the end of the intervention might have been related to the demyelination process that is characteristic of patients with MS, which alters VSR [64]. Also, the general reduction of muscular tone found in the aforementioned muscles may be explained by better balance performance and decreasing VSR activity, according to Forbes et al [65,66].

Lubetzky et al [67] declared acceptable usability (73% in the SUS) for the forerunner of the Oculus Quest, as compared to 90% usability reported by our participant. Furthermore, that author confirmed that the existence of wires reduced immersion and the presence of the users within the VRi environment. This problem is solved with the wireless Oculus Quest HMD. Additionally, thanks to the selected HMD and the design of our vestibular exercises, intrinsic Cawthorne-Cooksey protocol limitations can be addressed. These limitations are overcome by adopting a multimodal approach, providing extrinsic feedback, task-oriented focus, and exposure to different environments [68,69]. Also, considering the VRi vestibular protocol design and the portable and wireless HMD device, one future field of research could be telerehabilitation strategies. This field of study is still poorly investigated regarding vestibular rehabilitation and VR.

### Limitations and Strengths

The main limitations of this study were derived from the study design; thus, selection bias may have been present, and it is not possible to establish cause-and-effect relationships nor to make general statements regarding the MS population. Another limitation would be the question as to whether this intervention would be favorable in all phenotypes of MS or in central, peripheral, or mixed vestibular disorders. Because of this, the results must be interpreted with caution. To provide additional scientific evidence, a randomized controlled trial will be performed.

A principal strength of this study is that it provides the first standardized VRi vestibular training protocol for an MS population. Thanks to the design of the exercises and the characteristics of the selected HMD, the limitations of the gold-standard Cawthorne-Cooksey protocol are overcome. This innovative VRi vestibular protocol was designed to allow its implementation at clinic, hospital, and home and as a telerehabilitation strategy. In addition to the expected effects of vestibular rehabilitation, this protocol shows benefits from VRi. Lastly, the selected exergames are freely available, which allows professionals who have HMD devices to implement this VRi vestibular protocol without additional costs.

### Conclusions

The first standardized VRi vestibular protocol based on a gold-standard protocol was carried out on a subject with RRMS. The protocol showed promising results for dizziness, postural balance, gait, fatigue, and quality of life after the experimental intervention, although the results should be interpreted with caution due to the design of the study. The intervention described in this case study could set a precedent for future VRi vestibular interventions for vestibular disorders, specifically in the MS population. To achieve solid results in relation to this innovative protocol, it is necessary for further research to be conducted, such as a randomized controlled trial. Another future approach that could evaluate the effects of this VRi vestibular intervention would be a telerehabilitation strategy.

## Abbreviations

BBS: Berg Balance Scale
DHI: Dizziness Handicap Inventory
HMD: head-mounted display
iTUG: instrumented Timed Up and Go
MFIS: Modified Fatigue Impact Scale
MRI: magnetic resonance imaging
MS: multiple sclerosis
MSQoL-54: Multiple Sclerosis Quality of Life-54
RRMS: relapsing-remitting multiple sclerosis
SUS: System Usability Scale
T0: baseline
T1: between initial and advanced phases
T2: postintervention
T3: 1 month after the experimental procedure
VOR: vestibulo-ocular reflex
VR: virtual reality
VRi: immersive virtual reality
VSR: vestibulo-spinal reflex
